# Accuracy of the direct agglutination test for diagnosis of visceral leishmaniasis: a systematic review and meta-analysis

**DOI:** 10.1186/s12879-023-08772-1

**Published:** 2023-11-09

**Authors:** Tamalee Roberts, Suzanne H. Keddie, Sayaphet Rattanavong, Santiago Rayment Gomez, John Bradley, Ruth H. Keogh, Oliver Bärenbold, Jane Falconer, Petra F. Mens, Heidi Hopkins, Elizabeth A. Ashley

**Affiliations:** 1 Lao- Oxford-Mahosot Hospital- Wellcome Trust Research Unit, Mahosot Hospital, Mahosot Road, Vientiane, Lao People’s Democratic Republic; 2 Centre for Tropical Medicine and Global Health, Nuffield Department of Medicine, University of Oxford, Oxford, UK; 3 London School of Hygiene & Tropical Medicine, London, UK; 4 Department of Infectious Disease Epidemiology, Faculty of Medicine, London Centre for Neglected Tropical Disease Research, St Marys Campus, Imperial College London, London, UK; 5 Department of Medical Microbiology, Experimental Parasitology Unit, Amsterdam University Medical Centers, Academic Medical Center at the University of Amsterdam, Amsterdam, The Netherlands

**Keywords:** Visceral leishmaniasis, Direct agglutination test, Diagnosis, *Leishmania*, Meta-analysis

## Abstract

**Background:**

Parasitological investigation of bone marrow, splenic or lymph node aspirations is the gold standard for the diagnosis of visceral leishmaniasis (VL). However, this invasive test requires skilled clinical and laboratory staff and adequate facilities, and sensitivity varies depending on the tissue used. The direct agglutination test (DAT) is a serological test that does not need specialised staff, with just minimal training required. While previous meta-analysis has shown DAT to have high sensitivity and specificity when using parasitology as the reference test for diagnosis, meta-analysis of DAT compared to other diagnostic techniques, such as PCR and ELISA, that are increasingly used in clinical and research settings, has not been done.

**Methods:**

We conducted a systematic review to determine the diagnostic performance of DAT compared to all available tests for the laboratory diagnosis of human VL. We searched electronic databases including Medline, Embase, Global Health, Scopus, WoS Science Citation Index, Wiley Cochrane Central Register of Controlled Trials, Africa-Wide Information, LILACS and WHO Global Index. Three independent reviewers screened reports and extracted data from eligible studies. A meta-analysis estimated the diagnostic sensitivity and specificity of DAT.

**Results:**

Of 987 titles screened, 358 were selected for full data extraction and 78 were included in the analysis, reporting on 32,822 participants from 19 countries. Studies included were conducted between 1987–2020. Meta-analysis of studies using serum and DAT compared to any other test showed pooled sensitivity of 95% (95%CrI 90–98%) and pooled specificity of 95% (95%CrI 88–98%). Results were similar for freeze-dried DAT and liquid DAT when analysed separately. Sensitivity was lower for HIV-positive patients (90%, CrI 59–98%) and specificity was lower for symptomatic patients (70%, CrI 43–89%). When comparing different geographical regions, the lowest median sensitivity (89%, CrI 67–97%) was in Western Asia (five studies).

**Conclusions:**

This systematic review and meta-analysis demonstrates high estimated pooled sensitivity and specificity of DAT for diagnosis of VL, although sensitivity and specificity were lower for different patient groups and geographical locations. This review highlights the lack of standardisation of DAT methods and preparations, and the lack of data from some important geographical locations. Future well-reported studies could provide better evidence to inform test implementation for different patient populations and use cases.

**PROSPERO registration:**

CRD42021240830

**Supplementary Information:**

The online version contains supplementary material available at 10.1186/s12879-023-08772-1.

## Background

Leishmaniasis is a vector-borne disease caused by the protozoan parasite of the genus *Leishmania.* The parasite is transmitted by the bite of female phlebotomine sand flies [1]. There are three main clinical forms of leishmaniasis: cutaneous leishmaniasis (CL) causing ulcerated skin lesions; mucocutaneous leishmaniasis (MCL), which can lead to partial or total destruction of mucous membranes; and visceral leishmaniasis (VL) which is a systemic, potentially lethal disease [2, 3]. There are an estimated 600,000 to 1 million new cases of CL and 50,000 to 90,000 new cases of VL reported annually [4]. As of 2021, there were 99 countries and territories endemic for leishmaniasis with 89% of global VL cases reported from eight countries: Brazil, Ethiopia, India, Kenya, Somalia, South Sudan, Sudan and Yemen [5]. VL has emerged as an opportunistic infection associated with HIV. People living with HIV are more likely to develop VL, and VL is an AIDS-defining condition due to both HIV and *Leishmania* suppressing the immune system which can result in more severe VL disease and higher mortality rates than from either infection in isolation [6].

Parasitological investigation of splenic, lymph node or bone marrow aspirates by microscopic examination for amastigotes remains the gold standard for the diagnosis of VL around the world with sensitivity ranging from 60–99% depending on the sample type [7]. These invasive tests require skilled clinical and laboratory staff and appropriate medical facilities meaning parasitological investigation is often not possible in leishmaniasis-endemic countries. Other diagnostic tests include the direct agglutination test (DAT), enzyme linked immunosorbent assay (ELISA), immunofluorescence antibody test (IFAT), immunochromographic tests, latex agglutination tests, leishmanin skin test (LST) and molecular techniques, including polymerase chain reaction (PCR). All of these tests use different sample types and have varying sensitivity and specificity but are more accessible within leishmaniasis-endemic countries.

DAT is a simple and reliable serological test for the diagnosis of VL which has been on the WHO’s list of essential in vitro diagnostics since 2021. DAT is a semi-quantitative test that uses V-shaped well microplates with stained killed promastigotes of *L. donovani* or *L. infantum* mixed with increasing dilutions of patient’s serum or blood*.* DAT detects the presence of antibodies against *Leishmania* parasites in the patient’s serum or blood. If antibodies are present these will form an agglutination complex with the promastigotes which can be seen as a blue thin film on the walls of the microplates. The results can be read after 18 h of incubation. A titre-cut off for a specific dilution is used to determine if the sample is positive or negative for *Leishmania* with different cut-off titres used in different settings. Freeze dried antigen DAT (FD-DAT) and liquid antigen DAT (LQ-DAT) are the most common methods used and are based on the methods developed by Harith et al. [8]. LQ-DAT was developed first, however due to batch-to-batch variability as well as temperature sensitivity, FD-DAT was developed which remains stable at higher temperatures and has a higher shelf life with early validation studies showing similar results [9]. However, there are several other types of DAT including fast agglutination screening test (FAST-DAT) which uses a single serum dilution (qualitative result), formaldehyde fixed antigen DAT (FF-DAT) and in-house produced aqueous antigen DAT (AQ-DAT). Promastigotes and FD-DAT kits are produced by the former Royal Tropical Institute (KIT) Amsterdam (now Academic Medical Centre (AMC), Amsterdam), the Netherlands and the Institute of Tropical Medicine in Antwerp (ITMA), Belgium but liquid DAT is also often produced locally in-house with local strains.

Previous meta-analysis of DAT compared to parasitological examination for patients with *L. donovani* and *L. infantum* from studies published from 1986 to 2004 showed a pooled sensitivity and specificity of 94.8% and 85.9% respectively [10], while for studies from 2004 to 2019 the pooled sensitivity and specificity were 96% and 95% respectively [11]. In HIV-positive patients, DAT compared to microscopy showed lower sensitivity using random effects models of 81% and a specificity of 90% [12]. While meta-analysis of DAT accuracy compared to parasitological tests has been reported, meta-analysis of DAT compared to other diagnostic techniques, which are increasing in use in clinical and research settings and in low- and middle- income country (LMIC) settings, has not been done. This makes comparison of different studies and burden estimation difficult. Therefore, the aim of this study was to carry out a systematic review and meta-analysis to assess the diagnostic accuracy of DAT for human VL compared to all available tests up to February 2021.

## Methods

### Selection criteria

Eligible studies included prospective and retrospective studies on the diagnosis of human VL, independent of study design, that reported results of DAT and at least one comparator test. Case studies with ≤ 5 people and studies on diseases other than VL were excluded. See Table 1 for full inclusion and exclusion criteria.

**Table 1:** Systematic review of the direct agglutination test (DAT) for the diagnosis of visceral leishmaniasis in humans: inclusion and exclusion criteria

**Inclusion criteria**	Prospective and retrospective studies on diagnosis of visceral leishmaniasis, independent of study design
	Any variation of the DAT technique and a comparator standard used for diagnosing visceral leishmaniasis
	Paired data: The same samples tested with any variation of the DAT method compared to any comparator standard
	Epidemiological and or laboratory studies
**Exclusion criteria**	Lack of data (studies that do not include, for example, individual participant results, comparator standard, *Leishmania* species/origin, study type, sample type; see also Fig. 1)
	Discrepancies suspected between the studied group and the control group
	Reviews
	Commentaries
	Case studies with ≤ 5 cases
	Duplicate publications
	Patients with others types of leishmaniasis or infectious diseases other than visceral leishmaniasis
	Patients with post kalazar dermal leishmaniasis
	Animal studies

### Literature search strategy

The review was reported according to the Preferred Reporting Items for Systematic Reviews and Meta-Analyses (PRISMA) statement [13] (Additional file 1) and is registered with the international prospective register of systematic reviews (PROSPERO CRD42021240830).

Search terms were developed and carried out in 10 databases: OvidSP Medline (1946 to 12 February 2021), OvidSP Embase Classic + Embase (1947 to 12 February 2021), OvidSP Global Health, (1910 to week 05 2021), Elsevier Scopus (complete database), Clarivate Analytics Web of Science Science Citation Index (1970-present), Clarivate Analytics Web of Science Social Sciences Citation Index (1970-present), Wiley Cochrane Central Register of Controlled Trials (Issue 2 of 12, February 2021), Ebsco Africa-Wide Information (complete database), WHO LILACS (complete database), WHO Global Index Medicus (complete database). All searches were run on 15 February 2021. There was no restriction on language (see Additional file 2 for search strategy).

All citations identified from the searches were imported into EndNote X9 software. Duplicates were identified and removed using the method described on the London School of Hygiene & Tropical Medicine Library & Archives Service blog [14]. Additional eligible studies were hand-searched from the reference list of relevant manuscripts.

### Study selection and full-text review

Two independent reviewers (SR, TR) screened all titles and abstracts, as well as full texts when the abstract did not provide sufficient information, for compliance with the inclusion and exclusion criteria. Results were compared and discrepancies discussed with a third reviewer (EA).

### Data extraction

Selected full articles were screened independently and data extracted by three reviewers (SR, TR and SRG). All data was checked by a second reviewer and discrepancies discussed. Variables included bibliographic information, sample type, study design, study location, study population, number of participants, HIV status, DAT test kit details, comparator test details, number of samples tested by each test, patient age group, cross-reaction and quality control information, number positive and negative by each test and 2 × 2 tables (DAT + /Comparator + , DAT-/Comparator + , DAT + /Comparator-, DAT-/Comparator-).

### Assessment of study quality

The quality of studies was assessed using the Quality Assessment of Studies of Diagnostic Accuracy Approach-2 (QUADAS-2) [15]. Studies were assessed in duplicate by three assessors (SR, TR and SRG) and results compared.

### Categorisation of tests

All studies irrespective of DAT type were included. Where possible, further grouping of DAT was done according to manufacturer (e.g. ITMA, KIT, AMC, In-house) but sub-group analysis was not performed due to the low number of studies for each manufacturer type. However, sub-group analysis was carried out for FD-DAT and LQ-DAT as they were the DAT types most commonly reported.

### Geographical classification of countries and study population

Countries were classified by geographic sub-region following United Nations designations [16]. Study populations were grouped into four categories: neonates (aged ≤ 28 days), infants (1 to < 12 months), children (1 to < 13 years), and adolescents/adults (≥ 13 years). If a study reported participants from each age group, they were grouped as participants of “all ages”.

### Statistical analyses

Data extracted from each study included the 2 × 2 table comparing results of the index test (DAT) and a comparator test. Where a study presented results of DAT and multiple comparator tests, a 2 × 2 table for each comparator test was extracted. Descriptive analysis was completed for all studies.

To summarise the data from this review and estimate the sensitivity and specificity of DAT, the study implemented an extension to the hierarchical summary receiver operating characteristic model [17] described by Dendukuri, et al. [18]. Using this Bayesian model framework allows estimation of the accuracy of a diagnostic test in the absence of gold-standard comparator tests while taking account of within- and between-study variability (for example, each study is assumed to use a different positivity threshold). Within this approach the assumption of conditional independence between an individual’s test results, given their disease status, can be relaxed through the use of random-effects [19].

The outputs of this review are the estimated accuracy (sensitivity and specificity) with credible intervals [CrI]) of DAT within each study, as well as a pooled and predicted estimate of the test’s accuracy across all studies included. Results are presented in forest plots and as summary receiver operating characteristic (SROC) curves. Pooled sensitivity and specificity represent the summary of DAT test accuracy across studies included in this review, while predicted sensitivity and specificity allow estimation of the accuracy of DAT in a hypothetical future study. Where there is variability among studies, predicted sensitivity and specificity are less precise than pooled sensitivity and specificity.

A meta-analysis was fit on all data from serum samples only due to it being the most common sample type and to limit the number of variables that may affect the accuracy of DAT and therefore to strengthen the analysis and interpretation of results, irrespective of the specific DAT test and comparator test. We investigate heterogeneity by DAT test type (FD-DAT or LQ-DAT), geographic region, participant status (e.g. symptomatic or HIV-positive) and whether or not the assumption of conditional independence is assumed. Where multiple 2 × 2 tables are available from the same study and represent the same individuals, only one table is included in the meta-analysis to prevent including individuals in the model more than once. In these cases, we chose to include the 2 × 2 table where the comparator test’s accuracy (sensitivity and/or specificity) was better established (the one with the smallest difference between the 2.5 and 97.5 percentiles of the proposed prior distribution) as this understanding of the comparator test could be included into the model as informative prior distributions. For example, if there was a 2 × 2 table between DAT and PCR as well as DAT and microscopy, the 2 × 2 table with microscopy was chosen.

All analyses were carried out in R using stan [20]. A full model specification can be found in Additional file 3. All code can be found at: https://github.com/shk313/diagnostic-test-metaanalysis/tree/main/Leishmaniasis.

## Results

### Search results

**Fig. 1:**
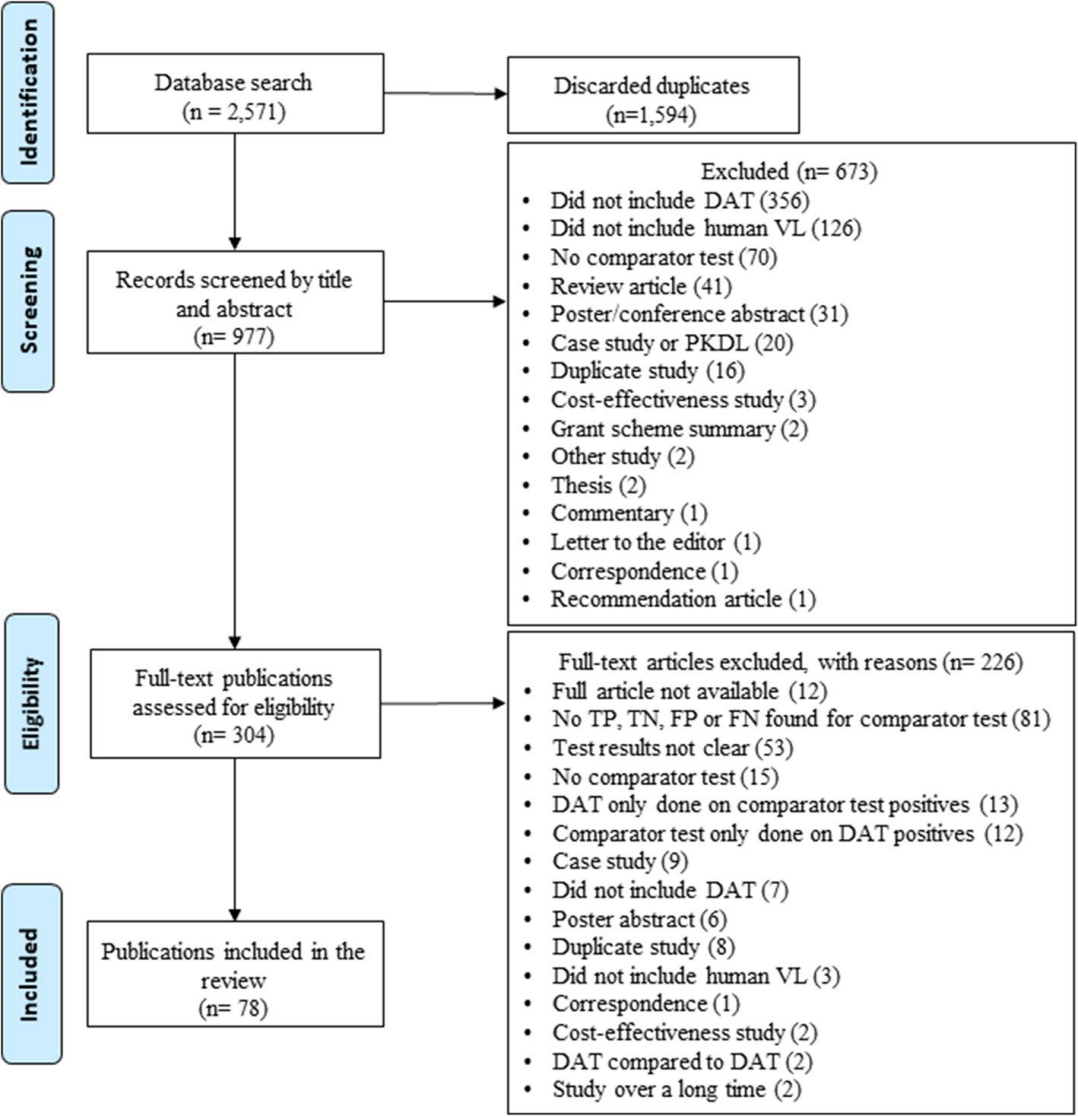
Preferred Reporting Items for Systematic Reviews and Meta-Analysis (PRISMA) flow diagram of publications screened in a systematic review of the direct agglutination test for the diagnosis of visceral leishmaniasis. DAT = Direct agglutination test; VL = Visceral leishmaniasis; PKDL = Post-kalazar dermal leishmaniasis; TP = True positive; TN = True negative; FP = False positive; FN = False negative

A total of 2571 articles were retrieved, 1584 of which were duplicates resulting in 987 titles and abstracts screened. Of these, 358 articles were selected for full data extraction and after full data extraction 78 articles were included that had complete 2 × 2 tables (Fig. 1).

### Study description

The included studies reported on a total of 32,822 patients from 19 countries. All patients were from countries endemic for leishmaniasis or had travelled to endemic countries. Ten studies included only adolescents/adults, four included only children, 47 included all age groups and 17 did not report participant ages. The studies included in this review were conducted from 1987 to 2020, inclusive.

Serum was tested in 63/78 (80.8%) studies, whole blood in 8/78 (10.3%), plasma in 5/78 (6.4%) and the sample type was not reported in 2/78 (2.6%). There was a range of DAT titre cut-offs with the lowest 1:100 and the highest ≥ 120,000. HIV-positive patients were included in 8/78 (10.3%) studies. Cross-reaction of DAT for VL with other diseases was noted in 8/78 (10.3%) studies, with cross-reaction noted for patients with cutaneous leishmaniasis (two studies), malaria (two studies), leukaemia (two studies), schistosomiasis (one study), Chagas disease (one study) and connective tissue disorder and lymphoma (one study). Symptomatic patients were included in 22/78 (28.2%) studies, asymptomatic in 9/78 (11.5%), both symptomatic and asymptomatic in 38/78 (48.7%) and it was not reported whether patients were symptomatic or not in 9/78 (11.5%) studies. Laboratory testing quality control was clearly stated in 22/78 (26.8%) studies. There were eight different types of DAT and 21 different comparator tests with the median number of comparator tests in included studies 1 (range 1–4).

### Meta-analysis sensitivity and specificity

**Fig. 2:**
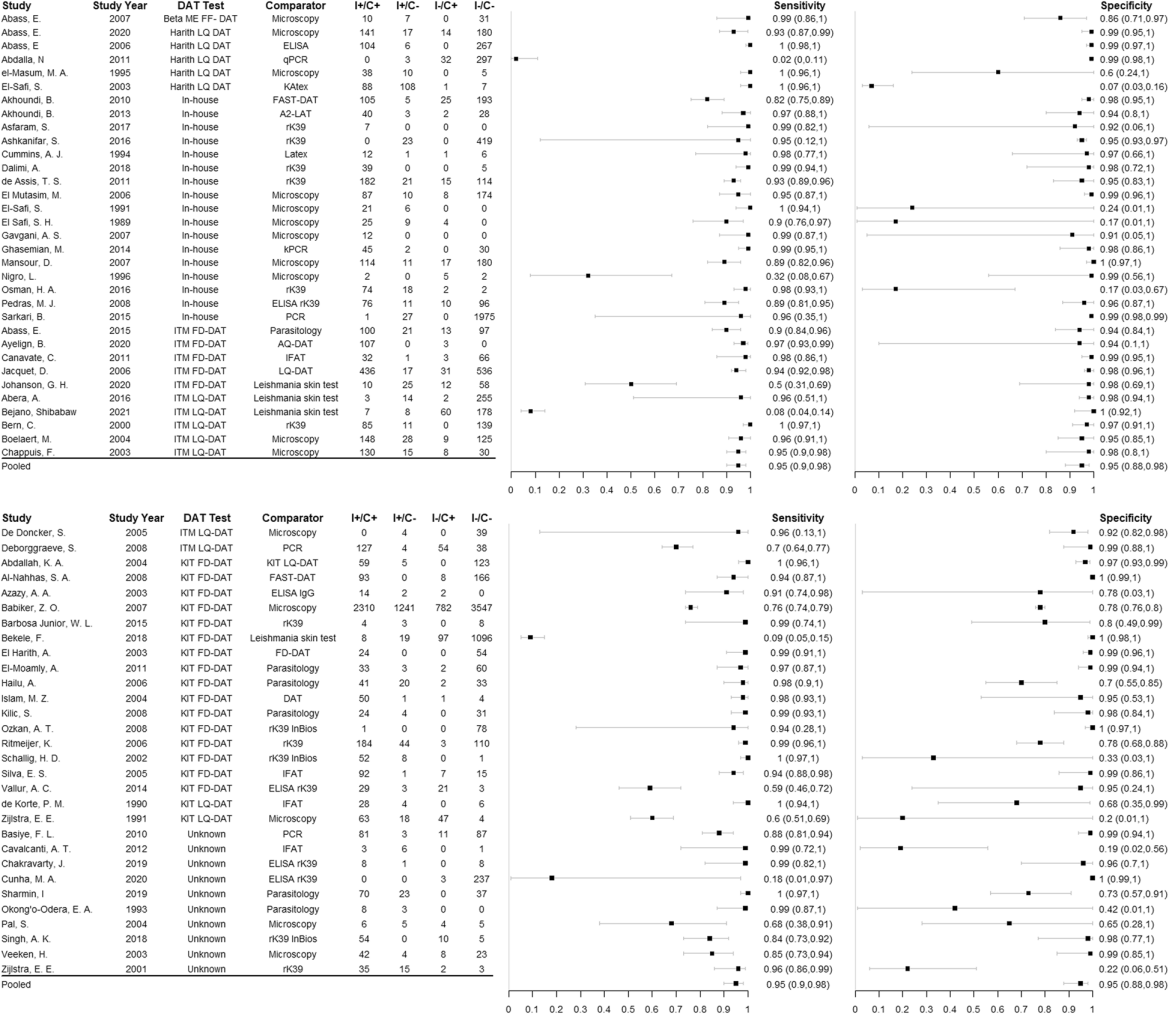
Forest plot of the sensitivity and specificity of DAT for the 63 studies that included serum sample type

**Fig. 3:**
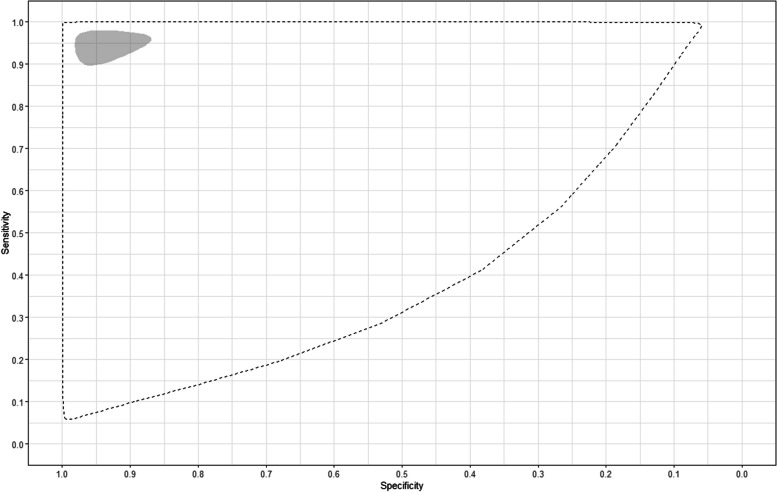
Summary receiver operating characteristic curves for pooled (shaded) and predicted (dashed) estimates of DAT for the 63 studies including serum sample type

Sixty-three studies representing 20,364 individuals that used serum samples and any DAT and comparator test were included in a meta-analysis; the results are shown in Fig. 2. The pooled sensitivity across all included studies was 95% (95% CrI 90–98%) and the pooled specificity across all included studies was 95% (95% CrI 88–98%). The estimated median sensitivity and specificity of DAT in included studies ranged from 2 to 100% and from 7 to 100% respectively. The predicted sensitivity and specificity were 96% (95% CrI 8–100%) and 96% (95% CrI 9–100%) respectively. The pooled and predicted estimates are shown as summary ROC curves in Fig. 3.

**Fig. 4:**
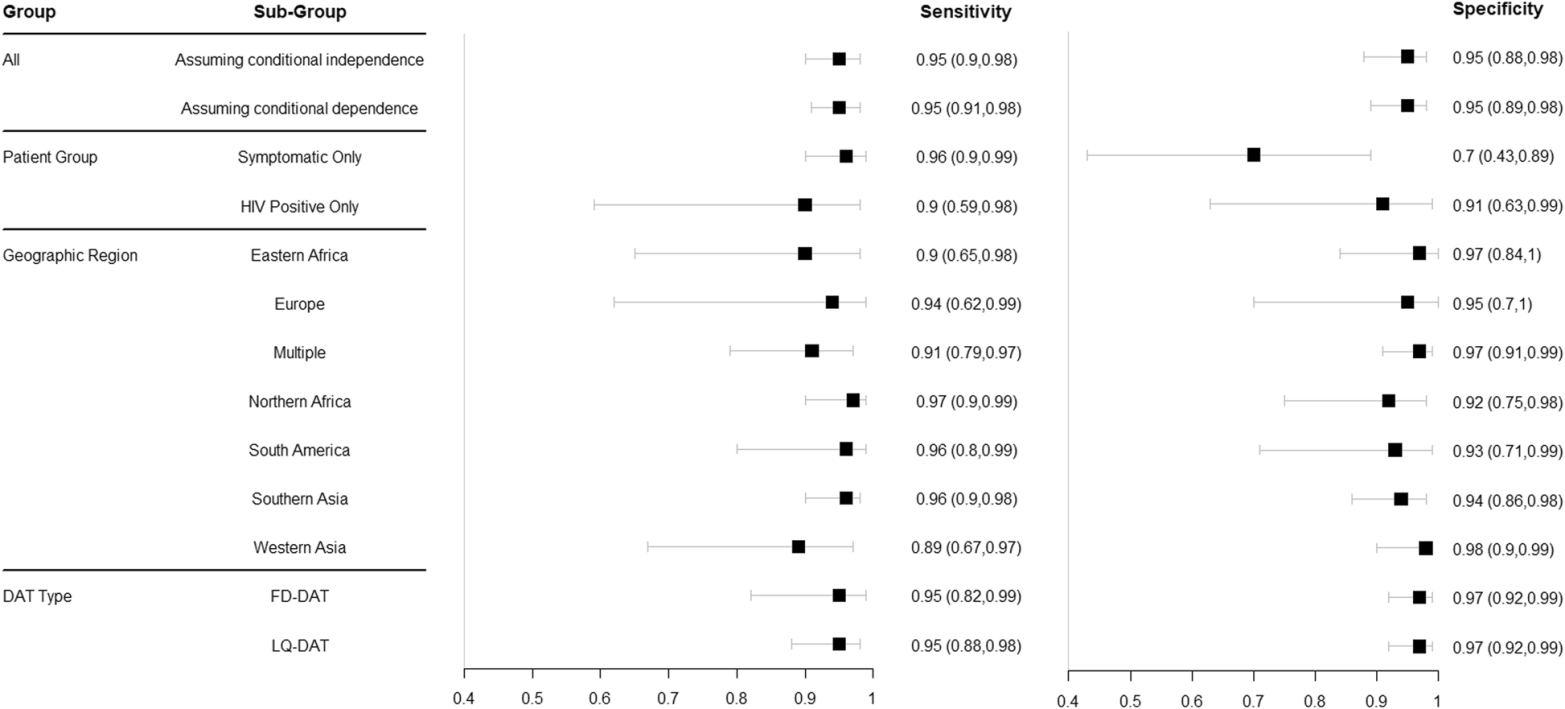
Forest plot of the pooled estimates of sensitivity and specificity grouped for all the data, studies from symptomatic patients, studies grouped by geographic region, and specific analysis for studies that include FD-DAT or LQ-DAT

**Fig. 5:**
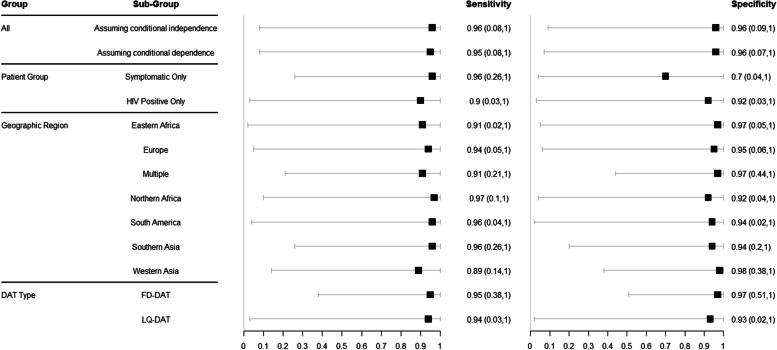
Forest plot of the predicted estimates of sensitivity and specificity grouped for all the data, studies from symptomatic patients, studies grouped by geographic region, and specific analysis for studies that include FD-DAT or LQ-DAT

We investigated heterogeneity in sensitivity and specificity estimates by patient group, geographic region, DAT test type and by relaxing the assumption of conditional independence between diagnostic tests within a study. The pooled estimates of DAT sensitivity and specificity from each analysis are shown in Fig. 4 and the predicted estimates are shown in Fig. 5. The number of studies and individuals included in each analysis are shown in Table 2. The pooled sensitivity estimates of DAT across these different analyses ranged from 89%-97% while the pooled specificity ranged from 70%-98%. Overall, from the studies included in this analysis, estimated DAT accuracy differed only slightly by geographical region. Western Asia (including five studies) had the lowest median sensitivity (89%, CrI 67–97%) and Europe (including two studies) was the region with the most uncertainty in sensitivity and specificity estimates. Pooled sensitivity and specificity were also similar when FD-DAT and LQ-DAT were analysed separately. Within the different patient groups, sensitivity and specificity estimates varied with a lower sensitivity and specificity with wider uncertainty for the HIV-positive patient group (90%, CrI 59–98% and 91%, CrI 63–99 respectively) and a low specificity for the symptomatic patient group (70%, CrI. 43–89%) compared to analyses that did not differentiate by patient group. The small number of studies that included HIV-positive patients, and the heterogeneity across these, precluded further investigation of accuracy among HIV-infected subgroups. Similarly, the small number of studies that included children, or distinguished between adult and child participants, precluded age-stratified analysis.

**Table 2:** The number of studies and individuals broken down for the meta-analysis for studies using specimen type serum

Model	Number of studies included	Number of Individuals represented
All	63	20,364
Symptomatic only	20	9266
HIV positive only	8	826
Geographic region: Eastern Africa	8	2248
Geographic region: Europe	2	47
Geographic region: Multiple	5	1537
Geographic region: Northern Africa	17	10,628
Geographic region: South America	7	966
Geographic region: Southern Asia	19	4220
Geographic region: Western Asia	5	521
DAT test: LQ	25	6094
DAT test: FD	19	12,273

The predicted estimates of DAT sensitivity and specificity by sub-group all have wide credible intervals because of heterogenous results from individual studies within a sub-group. The analysis considering only FD-DAT had the narrowest predicted credible intervals at 95% (CrI 38–100%) and 97% (CrI 51–100%) for sensitivity and specificity respectively, representing the DAT type with the least variability between studies.

### Assessment of study quality

Additional file 4 summarises the QUADAS-2 risk of bias and applicability concerns for the final included studies. Full information was not available for one study so it was not included in the QUADAS-2 analysis. For patient selection 39/77 (50.6%) studies had a high risk of bias, 7/77 (9.1%) had a high risk of bias for the index test, all had a low risk of bias for the comparator test and 4/77 (5.2%) had a high risk of bias for the flow and timing. All studies had low concern for applicability for patient selection, index test and comparator test.

## Discussion

This review evaluated the sensitivity and specificity of DAT, compared with all other available tests, for detecting human VL. We included 63 studies in a meta-analysis and found a high pooled sensitivity for any DAT of 95% (95% CrI 90–98%) and a pooled specificity of 95% (95% CrI 88–98%). This is similar to previous reviews that looked specifically at DAT compared to parasitological examination [10, 11]. We found little variability between geographic regions or DAT test type but sensitivity and specificity did vary when used in only symptomatic or HIV-positive patients.

A number of different DAT types were included in the initial analysis for serum samples with different antigen preparations and methods, and antigens may have been made on site or ordered from a manufacturer. Due to the number of different DAT preparation types, it was not possible to do sub-analysis on all distinct test types. The different DAT preparation types may impact real-world results, however, and future studies may be needed to compare the different preparation types. In this analysis, we found that the predicted estimates of sensitivity and specificity for FD-DAT had narrower credible intervals compared to LQ-DAT when analysed separately, suggesting that FD-DAT would be a more appropriate test.

We estimated the specificity of DAT to be lower when used in studies that enrolled only symptomatic patients, compared with studies that included any patient type (i.e. studies including both symptomatic and asymptomatic patients or those of unknown status). This finding aligns with a previous meta-analysis of DAT compared to microscopy where the lowest specificity was seen in patients clinically suspected to have VL [10]. Cross-reaction of both the FD-DAT and LQ-DAT with other diseases was reported in eight studies. This cross-reaction may have been one cause for the lower specificity of DAT for symptomatic patients. Cross-reaction is also something that laboratories and clinicians need to be aware of especially when testing patients from countries endemic for diseases such as malaria and other parasitic diseases. However, the reporting of patient selection was a concern for bias in 50% of included studies so these results should be treated with caution. Future studies should report clear patient selection criteria so that sub-group analysis can be carried out.

HIV-positive or otherwise immunocompromised patients frequently have low or undetectable anti-leishmanial antibodies meaning there is the potential for false-negative results from serological tests like DAT [12, 21]. Results from this review support this as a sub-analysis of studies with only HIV-positive patients showing a lower sensitivity and specificity with wider credible intervals than when all patients were included. As a result, studies using serological tests for the diagnosis of VL in HIV-positive patients should not rule out infection with a single negative test result.

Geographical region had some impact on pooled estimated sensitivity when FD-DAT and LQ-DAT were combined, with lower sensitivity seen for Western Asia. However, there were only five studies and 521 patients from Western Asia. The geographical variation was also seen in a previous review where sensitivity was higher in South Asia compared to other regions [10]. The sensitivity and specificity for the various DAT types and geographical regions should be taken into consideration by public health decision-makers when implementing diagnostic tests and further country-specific analysis should be done. Unfortunately, in this review there was not enough country-specific data to do focused country analysis and some regions were also lacking data (Europe and Western Asia). For some regions, even when multiple studies were available, data came from only one or two countries; more representative data would help to confirm if there is spatial heterogeneity in test accuracy. The species of promastigotes used in the DAT preparation may also affect test sensitivity and specificity, depending on their match with actual circulating *Leishmania* species in the region of interest.

Only 26% of studies clearly stated whether VL testing was subject to quality control, e.g. by testing samples at a reference laboratory and stating the positive and negative controls for the DAT. While most laboratories probably use controls and carry out quality control, stating this in the methods gives confidence to readers that laboratories’ results meet a recognised standard. For many studies, it was difficult to interpret the results due to a lack of clarity in reporting positive and negative results for each test. This review included studies published both before and after the introduction of STROBE (2007), STARD (2015) and MICRO (2019) reporting guidelines; even studies published after introduction did not adhere to the guidelines. Studies reporting laboratory and diagnostic test comparisons should report results to a standardised format, for consistency and comparability.

There are several limitations to this systematic review and meta-analysis. While we tried to be comprehensive and included studies comparing DAT to any other test, this identified reports on a wide range of DAT and comparator tests performed on a variety of sample types in a variety of geographical regions. This diversity may not have been fully captured by the analysis, and as a result estimates of DAT test accuracy may be biased in either direction. Despite this, our results are in line with other published estimates from studies with more restrictive selection criteria [8, 9]. A second limitation is that only a single 2 × 2 table from each study was included in the meta-analysis and the selection of which 2 × 2 table to include was somewhat subjective. Finally, different titre cut-offs were used across studies included in this review, but this was not accounted for in analysis due to the large number of different cut-offs used; further analyses that incorporate titre cut-offs would help to improve estimates of DAT accuracy. The different titre cut-offs used in this analysis may impact the sensitivity and specificity with lower cut-offs potentially resulting in false positives and higher titre cut-offs resulting in false negatives.

Strengths of this review include the rigorous and comprehensive approach which included 78 studies representing 32,822 individuals across 19 countries endemic for VL. Another strength is our analysis framework which did not assume any comparator test was perfect and which estimated both pooled and predicted sensitivity and specificity.

## Conclusion

Despite variability across studies in terms of geographic location, patient characteristics and comparator tests used, overall this systematic review and meta-analysis demonstrates that DAT performs well compared to other diagnostic methods in most scenarios. However, the test is generally not standardised with many methods and preparations of DAT in use. There is also a lack of data on DAT performance outside of South Asia and Northern Africa, with no data from Southeast Asia. Future studies carried out in a variety of locations with well documented DAT preparations are required to improve estimates of the DAT accuracy, and to better inform implementation for different patient populations and use cases.

### Supplementary Information

**Additional file 1.** Preferred Reporting Items for Systematic Reviews and Meta-Analyses (PRISMA) statement.

**Additional file 2.** What is the accuracy of the direct agglutination test (DAT) for the diagnosis of visceral leishmaniasis in humans? Search methodology.

**Additional file 3.** *Leishmania* DAT Review Model Specification.

**Additional file 4.** QUADAS-2 scoring for each study. Studies were scored as high or low risk of bias and applicability.

## Data Availability

The datasets supporting the conclusions of this article are included within the article and additional files and available in the github repository (https://github.com/shk313/diagnostic-test-metaanalysis/tree/main/Leishmaniasis).

## Abbreviations

AMC: Academic Medical Centre
AQ-DAT: Aqueous antigen direct agglutination test
CL: Cutaneous leishmaniasis
CrI: Credible interval
DAT: Direct agglutination test
ELISA: Enzyme linked immunosorbent assay
FAST-DAT: Fast agglutination screening test direct agglutination test
FD-DAT: Freeze dried antigen direct agglutination test
FF-DAT: Formaldehyde fixed antigen direct agglutination test
HIV: Human immunodeficiency virus
IFAT: Immunofluorescence antibody test
ITMA: Institute of Tropical Medicine in Antwerp
KIT: Royal Tropical Institute
LQ-DAT: Liquid antigen direct agglutination test
LST: Leishmanin skin test
MCL: Mucocutaneous leishmaniasis
PCR: Polymerase chain reaction
QUADAS-2: Quality Assessment of Studies of Diagnostic Accuracy Approach- 2
RDT: Rapid diagnostic test
SROC: Summary receiver operating characteristic
VL: Visceral leishmaniasis
