# Supplementary material for: Accuracy of the direct agglutination test for diagnosis of visceral leishmaniasis: a systematic review and meta-analysis
Source: BMC Infect Dis. 2023 Nov 9;23:782. doi: 10.1186/s12879-023-08772-1 (PMC10636880; doi:10.1186/s12879-023-08772-1)
Supplement: Supplementary file 2 — Additional file 2. What is the accuracy of the direct agglutination test (DAT) for the diagnosis of visceral leishmaniasis in humans? Search methodology. [file 12879_2023_8772_MOESM2_ESM.docx]

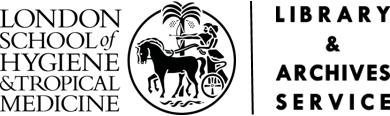


FIEBRE Reviews: What is the accuracy of the direct agglutination test (DAT) for the diagnosis of visceral leishmaniasis in humans?

Search methodology

Table of Contents

[1 Search methodology 2](#_Toc66868049)

[2 Databases 2](#_Toc66868050)

[3 Information management 3](#_Toc66868051)

[4 Results 3](#_Toc66868052)

[5 References 3](#_Toc66868053)

[6 Appendix: Search strategies 4](#_Toc66868054)

[6.1 OvidSP Medline 4](#_Toc66868055)

[6.2 OvidSP Embase 5](#_Toc66868056)

[6.3 OvidSP Global Health 7](#_Toc66868057)

[6.4 Wiley Cochrane CENTRAL database 8](#_Toc66868058)

[6.5 Clarivate Analytics Web of Science 9](#_Toc66868059)

[6.6 Ebsco Africa-Wide Information 10](#_Toc66868060)

[6.7 Scopus 11](#_Toc66868061)

[6.8 LILACS 12](#_Toc66868062)

[6.9 Global Index Medicus 13](#_Toc66868063)

# Search methodology

A draft search strategy was compiled in the OvidSP Medline database by an experienced information specialist (JF). The search strategy included strings of terms, synonyms and controlled vocabulary terms (where available) to reflect two concepts:

Concept 1: leishmaniasis

Concept 2: direct agglutination tests

As recommended in the Cochrane Handbook for Systematic Reviews of Diagnostic Test Accuracy, a specific filter to identify diagnostic test accuracy studies was not included.^1^ Animal studies were excluded using the relevant section of the Cochrane RCT filter.^2^ No limits were applied. This search strategy was refined with the project team until the results retrieved reflected the scope of the project. The agreed OvidSP Medline search was adapted for each database to incorporate database-specific syntax and controlled vocabularies. Full details of the search strings used for each database can be found in the appendix.

# Databases

The following bibliographic databases were searched on 15 February 2021.

- OvidSP Medline ALL, 1946 to 12 February 2021
- OvidSP Embase Classic+Embase, 1947 to 12 February 2021.
- OvidSP Global Health, 1910 to week 05 2021.
- Wiley Cochrane Central Register of Controlled Trials, Issue 2 of 12, February 2021.
- Clarivate Analytics Web of Science, Data last updated 12 February 2021:
  - Science Citation Index-Expanded, 1970-present;
  - Social Sciences Citation Index, 1970-present.
- Elsevier Scopus, complete database.
- Ebsco Africa-Wide Information, complete database.
- WHO LILACS, complete database.
- WHO Global Index Medicus, complete database.

# Information management

All citations identified by our searches were imported into EndNote X9 software. Duplicates were identified and removed using the method described on the London School of Hygiene & Tropical Medicine Library & Archives Service blog.^3^

# Results

A total of 2571 results were retrieved by the search. 1584 (62%) were identified as duplicates. Number of results pre-and post-deduplication are listed in the table below.

| Database name | Total number of results | Number of results once duplicates removed |
| --- | --- | --- |
| Medline | 373 | 328 |
| Embase | 588 | 307 |
| Global Health | 416 | 110 |
| Africa-Wide Information | 129 | 3 |
| Cochrane Central Register of Controlled Trials | 14 | 1 |
| Global Index Medicus | 97 | 17 |
| Web of Science databases (both searched together) | 520 | 199 |
| Scopus | 400 | 11 |
| LILACS | 34 | 11 |
| **Total** | **2571** | **987** |

# References

1. de Vet HCW, Eisinga A, Riphagen II, Aertgeerts B, Pewsner D. Searching for Studies. Cochrane Handbook for Systematic Reviews of Diagnostic Test Accuracy: The Cochrane Collaboration; 2008. Available from: <https://methods.cochrane.org/sites/methods.cochrane.org.sdt/files/public/uploads/Chapter07-Searching-%28September-2008%29.pdf>

2. Lefebvre C, Glanville J, Briscoe S, Littlewood A, Marshall C, Metzendorf MI, et al. Technical Supplement to Chapter 4: Searching for and selecting studies. In: Higgins JPT, Thomas J, Chandler J, Cumpston MS, Li T, Page MJ, et al., editors. Cochrane Handbook for Systematic Reviews of Interventions 6th ed: Cochrane; 2019. Available from: <https://training.cochrane.org/handbook/version-6/chapter-4-tech-suppl>.

3. Falconer J. Removing duplicates from an EndNote Library. Library & Archives Service Blog [Internet]: London School of Hygiene & Tropical Medicine. 2018. [cited 2020]. Available from: <https://blogs.lshtm.ac.uk/library/2018/12/07/removing-duplicates-from-an-endnote-library/>.

# Appendix: Search strategies

This appendix provides full details of all search strings used for bibliographic databases, with dates and number of references returned and notes explaining any unusual search techniques or syntax. The EndNote X9 import order is provided, as the deduplication technique keeps the first uploaded copy of the reference by default.

In all searches, numbers in parentheses at the end of each row show the number of hits retrieved.

## OvidSP Medline

| Database name | Medline ALL |
| --- | --- |
| Database platform | OvidSP |
| Dates of database coverage | 1946 to 12 February 2021 |
| Date searched | 15 February 2021 |
| Searched by | JF |
| Number of results | 373 |
| EndNote import order | 1 |
| Number of results once duplicates removed | 328 |
| Search strategy notes | Search lines ending in a ‘/’ are subject heading searches. Search lines beginning ‘exp’ are exploded subject heading searches. Two-letter codes at the end of search lines designate the fields to search. Fields codes used are: TI: title AB: abstract SH: subject heading or/*x-y* combines search sets in the range *x-y* with Boolean operator OR. * is used for truncation of words. |

1. leishmaniasis/ or leishmaniasis, visceral/ (16522)
2. leishmania/ or leishmania donovani/ or leishmania infantum/ (15058)
3. (leishmaniasis or kala-azar).ti,ab. (23890)
4. (leishmania or "l donovani" or "l chagasi" or "l infantum" or "l martiniquensis" or "l siamensis").ti,ab. (25346)
5. or/1-4 (38204)
6. Agglutination Tests/ (11807)
7. direct agglutination test*.ti,ab. (569)
8. fast agglutination screening test.ti,ab. (14)
9. (DAT or FAST-DAT or DAT-LPC).ti,ab. (8031)
10. or/6-9 (19753)
11. 5 and 10 (463)
12. exp animals/ not humans.sh. (4787759)
13. 11 not 12 (373)
14. limit 13 to medline (318)
15. 13 not 14 (55)

## OvidSP Embase

| Database name | Embase Classic+Embase |
| --- | --- |
| Database platform | OvidSP |
| Dates of database coverage | 1947 to 12 February 2021 |
| Date searched | 15 February 2021 |
| Searched by | JF |
| Number of results | 588 |
| EndNote import order | 2 |
| Number of results once duplicates removed | 307 |
| Search strategy notes | Search lines ending in a ‘/’ are subject heading searches. Search lines beginning ‘exp’ are exploded subject heading searches. Two-letter codes at the end of search lines designate the fields to search. Fields codes used are: TI: title AB: abstract SH: subject heading or/*x-y* combines search sets in the range *x-y* with Boolean operator OR. * is used for truncation of words. |

1. leishmaniasis/ or visceral leishmaniasis/ (25352)
2. leishmania/ or leishmania chagasi/ or leishmania donovani/ (17544)
3. (leishmaniasis or kala-azar).ti,ab. (28149)
4. (leishmania or "l donovani" or "l chagasi" or "l infantum" or "l martiniquensis" or "l siamensis").ti,ab. (29335)
5. or/1-4 (47662)
6. agglutination test/ (15559)
7. direct agglutination test*.ti,ab. (687)
8. fast agglutination screening test.ti,ab. (15)
9. (DAT or FAST-DAT or DAT-LPC).ti,ab. (12184)
10. or/6-9 (27482)
11. 5 and 10 (634)
12. (rat or rats or mouse or mice or swine or porcine or murine or sheep or lambs or pigs or piglets or rabbit or rabbits or cat or cats or dog or dogs or cattle or bovine or monkey or monkeys or trout or marmoset$1).ti. and animal experiment/ (1102932)
13. Animal experiment/ not (human experiment/ or human/) (2322812)
14. 12 or 13 (2375283)
15. 11 not 14 (598)
16. remove duplicates from 15 (588)

## OvidSP Global Health

| Database name | Global Health |
| --- | --- |
| Database platform | OvidSP |
| Dates of database coverage | 1910 to week 05 2021 |
| Date searched | 15 February 2021 |
| Searched by | JF |
| Number of results | 416 |
| EndNote import order | 3 |
| Number of results once duplicates removed | 110 |
| Search strategy notes | Search lines ending in a ‘/’ are subject heading searches. Search lines beginning ‘exp’ are exploded subject heading searches. Two-letter codes at the end of search lines designate the fields to search. Fields codes used are: TI: title AB: abstract SH: subject heading or/*x-y* combines search sets in the range *x-y* with Boolean operator OR. * is used for truncation of words. |

1. leishmaniasis/ or visceral leishmaniasis/ (30132)
2. leishmania/ or leishmania donovani/ or leishmania infantum/ (40971)
3. (leishmaniasis or kala-azar).ti,ab. (28138)
4. (leishmania or "l donovani" or "l chagasi" or "l infantum" or "l martiniquensis" or "l siamensis").ti,ab. (29295)
5. or/1-4 (42498)
6. agglutination tests/ (5781)
7. direct agglutination test*.ti,ab. (675)
8. fast agglutination screening test.ti,ab. (12)
9. (DAT or FAST-DAT or DAT-LPC).ti,ab. (873)
10. or/6-9 (6636)
11. 5 and 10 (500)
12. ((rat or rats or mouse or mice or swine or porcine or murine or sheep or lambs or pigs or piglets or rabbit or rabbits or cat or cats or dog or dogs or cattle or bovine or monkey or monkeys or trout or marmoset$1) not man).sh. (513012)
13. 11 not 12 (418)
14. remove duplicates from 13 (416)

## Wiley Cochrane CENTRAL database

| Database name | Cochrane Central Register of Controlled Trials |
| --- | --- |
| Database platform | Wiley |
| Dates of database coverage | Issue 2 of 12, February 2021 |
| Date searched | 15 February 2021 |
| Searched by | JF |
| Number of results | 14 |
| EndNote import order | 7 |
| Number of results once duplicates removed | 1 |
| Search strategy notes | * is used for truncation.  Searches ending :ti,ab,kw search the title, abstract and keywords.  Note, numbers in parentheses are results across all Cochrane databases. |

#1 MeSH descriptor: [Leishmaniasis] this term only (127)

#2 MeSH descriptor: [Leishmaniasis, Visceral] this term only (161)

#3 MeSH descriptor: [Leishmania] this term only (25)

#4 MeSH descriptor: [Leishmania donovani] this term only (33)

#5 MeSH descriptor: [Leishmania infantum] this term only (8)

#6 (leishmaniasis or "kala-azar"):ti,ab,kw (856)

#7 (leishmania or "l donovani" or "l chagasi" or "l infantum" or "l martiniquensis" or "l siamensis"):ti,ab,kw (342)

#8 #1 or #2 or #3 or #4 or #5 or #6 or #7 (869)

#9 MeSH descriptor: [Agglutination Tests] this term only (38)

#10 ("direct agglutination test*"):ti,ab,kw (12)

#11 ("fast agglutination screening test*"):ti,ab,kw (0)

#12 (DAT or "FAST-DAT" or "DAT-LPC"):ti,ab,kw (8545)

#13 #9 or #10 or #11 or #12 (8590)

#14 #8 and #13 (20)

## Clarivate Analytics Web of Science

| Database name | Science Citation Index Expanded Social Sciences Citation Index |
| --- | --- |
| Database platform | Clarivate Analytics Web of Science |
| Dates of database coverage | Both databases 1970-present. Data last updated 12 February 2021 |
| Date searched | 15 February 2021 |
| Searched by | JF |
| Number of results | 520 |
| EndNote import order | 5 |
| Number of results once duplicates removed | 199 |
| Search strategy notes | * is used for truncation.  TOPIC and TS searches search in the title, abstract and keywords fields.  TI searches search in the title.  All searches run across Indexes=SCI-EXPANDED, SSCI. Timespan=All years |

#1 TOPIC: (leishmaniasis or "kala-azar" or leishmania or "l donovani" or "l chagasi" or "l infantum" or "l martiniquensis" or "l siamensis") (40,953)

#2 TOPIC: ("direct agglutination test*" or "fast agglutination screening test*" or dat or "fast-dat" or "dat-lpc") (10,184)

#3 #2 AND #1 (547)

#4 TI=(rat or rats or mouse or mice or swine or porcine or murine or sheep or lambs or pigs or piglets or rabbit or rabbits or cat or cats or dog or dogs or cattle or bovine or monkey or monkeys or trout or marmoset*) NOT TS=(man or human) (2,299,790)

#5 #3 not #4 (520)

## Ebsco Africa-Wide Information

| Database name | Africa-Wide Information |
| --- | --- |
| Database platform | Ebsco |
| Dates of database coverage | Complete database as of search date |
| Date searched | 15 February 2021 |
| Searched by | JF |
| Number of results | 129 |
| EndNote import order | 4 |
| Number of results once duplicates removed | 3 |
| Search strategy notes | Two-letter codes at the beginning of search lines designate the fields to search. Fields codes used are: TI: title AB: abstract KW: keywords * is used for truncation of words. N*n* searches for terms within *n* words of each other. |

S1 (TI (leishmaniasis or "kala-azar")) OR (AB (leishmaniasis or "kala-azar")) (4,210)

S2 (TI (leishmania or "l donovani" or "l chagasi" or "l infantum" or "l martiniquensis" or "l siamensis")) or (AB (leishmania or "l donovani" or "l chagasi" or "l infantum" or "l martiniquensis" or "l siamensis")) (3,246)

S3 S1 OR S2 (5,524)

S4 (TI "direct agglutination test*") or (AB "direct agglutination test*") (146)

S5 (TI "fast agglutination screening test*") or (AB "fast agglutination screening test*") (3)

S6 (TI (DAT or FAST-DAT or DAT-LPC)) OR (AB (DAT or FAST-DAT or DAT-LPC)) (2,784)

S7 S4 OR S5 OR S6 (2,828)

S8 S3 AND S7 (136)

S9 (TI (rat or rats or mouse or mice or swine or porcine or murine or sheep or lambs or pigs or piglets or rabbit or rabbits or cat or cats or dog or dogs or cattle or bovine or monkey or monkeys or trout or marmoset*)) (84,025)

S10 (TI (man or human)) OR (AB (man or human)) OR (KW (man or human)) (514,922)

S11 S9 not S10 (74,036)

S12 S8 not S11 (129)

## Scopus

| Database name | Scopus |
| --- | --- |
| Database platform | Scopus.com |
| Dates of database coverage | Complete database as of search date |
| Date searched | 15 February 2021 |
| Searched by | JF |
| Number of results | 400 |
| EndNote import order | 6 |
| Number of results once duplicates removed | 11 |
| Search strategy notes | * is used for truncation of words.  { } searches for exact term with no lemmatization or stemming. |

(TITLE-ABS-KEY ({leishmaniasis} or "kala-azar" or {leishmania} or "l donovani" or "l chagasi" or "l infantum" or "l martiniquensis" or "l siamensis") AND TITLE-ABS-KEY ("direct agglutination test*" or "fast agglutination screening test*" or dat or "fast-dat" or "dat-lpc") ) AND NOT ((TITLE ({rat} OR {rats} OR {mouse} OR {mice} OR {swine} OR {porcine} OR {murine} OR {sheep} OR {lambs} OR {pigs} OR {piglets} OR {rabbit} OR {rabbits} OR {cat} OR {cats} OR {dog} OR {dogs} OR {cattle} OR {bovine} OR {monkey} OR {monkeys} OR {trout} OR marmoset*)) AND NOT (TITLE-ABS-KEY ({man} OR {human})))

## LILACS

| Database name | LILACS |
| --- | --- |
| Database platform | WHO Global Index Medicus |
| Dates of database coverage | Complete database as of search date |
| Date searched | 15 February 2021 |
| Searched by | JF |
| Number of results | 34 |
| EndNote import order | 8 |
| Number of results once duplicates removed | 11 |
| Search strategy notes | * is used for truncation of words. |

(tw:(leishmaniasis or "kala-azar" or leishmania or "l donovani" or "l chagasi" or "l infantum" or "l martiniquensis" or "l siamensis")) AND (tw:("direct agglutination test*" or "fast agglutination screening test*" or dat or "fast-dat" or "dat-lpc"))

## Global Index Medicus

| Database name | Global Index Medicus |
| --- | --- |
| Database platform | WHO Global Index Medicus |
| Dates of database coverage | Complete database as of search date |
| Date searched | 15 February 2021 |
| Searched by | JF |
| Number of results | 97 |
| EndNote import order | 9 |
| Number of results once duplicates removed | 17 |
| Search strategy notes | * is used for truncation of words. |

(tw:(leishmaniasis or "kala-azar" or leishmania or "l donovani" or "l chagasi" or "l infantum" or "l martiniquensis" or "l siamensis")) AND (tw:("direct agglutination test*" or "fast agglutination screening test*" or dat or "fast-dat" or "dat-lpc"))
