# Supplementary material for: Accuracy of the direct agglutination test for diagnosis of visceral leishmaniasis: a systematic review and meta-analysis
Source: BMC Infect Dis. 2023 Nov 9;23:782. doi: 10.1186/s12879-023-08772-1 (PMC10636880; doi:10.1186/s12879-023-08772-1)
Supplement: Supplementary file 3 — Additional file 3. Leishmania DAT Review Model Specification. [file 12879_2023_8772_MOESM3_ESM.docx]

**Additional file 3- *Leishmania* DAT Review Model Specification**

The complete model to estimate the sensitivity and specificity of the index test in each study, and hence predict sensitivity and specificity in a future study, consists of two hierarchical levels. The first level captures between-study variations in the sensitivity and specificity through a Hierarchical Summary Receiver Operating Characteristic (HSROC) model (1), which assumes that sensitivity and specificity across studies lie on an ROC curve. The second level accounts for the unknown true disease status of all participants and potential conditional dependence between diagnostic tests through the use of a random-effect latent class model (2).

Starting with the latent class model, assume we have a sample of N individuals who have all undergone two different dichotomous tests defined by $T_{r} (r =1,2), and let t_{ri}$ be a random variable denoting the outcome from test $r$for individual $i, i=1,..,N$. A positive test result for an individualis denoted by $t_{ri}=1$, and a negative test result by $t_{ri}=0.$ Also assume that the unknown true disease status of an individual, denoted $D$, can take one of two values: ‘diseased’ ($D=1)$ or ‘non-diseased’ ($D=0)$.The true (latent) disease status of the $i$^th^ individual is denoted $d_{i} .$ Assuming conditional independence between the two tests, we can write the joint distribution as:

| $\Pr\left( T_{1}= t_{1},T_{2}=t_{2} \right)=$  $Pr(T_{1}\vert D=1)Pr(T_{2}\vert D=1)Pr(D=1)+Pr(T_{1}\vert D=0)Pr(T_{2}\vert D=0)Pr(D=0)$ | 1 |
| --- | --- |

For the *r^t^*^h^ test, the sensitivity $(Se)$ and specificity $(Sp)$ can be written as $\mathrm{Se}_{r}=Pr(T_{r} =1|D=1)$ and$\mathrm{Sp}_{r}=Pr(T_{r}=0|D=0)$ respectively. We can also define prevalence as $\pi=Pr(D=1)$. We can then specify equation 1 in terms of sensitivity, specificity and prevalence:

| $\Pr\left( T_{1}= t_{1},T_{2}=t_{2} \right)=$  $\mathrm{Se}_{1}\mathrm{Se}_{2}(1-\mathrm{Se}_{1})(1-\mathrm{Se}_{2})\pi+(1-\mathrm{Sp}_{1})(1-\mathrm{Sp}_{2})\mathrm{Sp}_{1}\mathrm{Sp}_{2}(1-\pi)$ | 2 |
| --- | --- |

Following this, we can then define the likelihood for this latent class model assuming conditional independence as:

| $L = \prod_{i=1}^{N} (\pi{\mathrm{Se}_{1}}^{t_{1i}}{\mathrm{Se}_{2}}^{t_{2i}}(1-{\mathrm{Se}_{1})}^{1-t_{1i}}(1-{\mathrm{Se}_{2})}^{1-t_{2i}})+((1-\pi){\mathrm{Sp}_{1}}^{1-t_{1i}}{\mathrm{Sp}_{2}}^{1-t_{2i}}(1-{\mathrm{Sp}_{1})}^{t_{1i}}(1-{\mathrm{Sp}_{2})}^{t_{2i}})$ | 3 |
| --- | --- |

We account for conditional dependence between tests in disease positive individuals ($D=1)$ by allowing the sensitivity from each test to depend on an individual level random effect $s_{i}$. We assume that the sensitivity in the conditionally dependent model takes the form (1):

| ${Pr(T_{\mathrm{ri}}= 1\vert D_{i}=1,) =Se}_{\mathrm{ri}} = g^{-1}(a_{rd=1} + b_{rd=1}s_{i})$ | 4 |
| --- | --- |

where $g\left( \cdot\right)$ is a link function. In this study we use the logit link, $g^{-1}(y) = 1/(1+e^{-y})$, $a_{rd=1}$ and $b_{rd=1}$ are unknown parameters to be estimated where b describes the strength of dependence between two tests and the random effect $s_{i}$ follows a standard normal distribution. The subject-specific random-effect $s_{i}$ represents some unobserved characteristic for example infection intensity, that indirectly creates dependence between tests.

The study-level latent class model (equation 3 and 4) is then linked to the between-study level using a HSROC model (equation 5) which models between study variations by assuming that test sensitivity and specificity lie on an ROC curve. In particular, each study $j$ $(j=1,..,J)$provides the 2x2 table between the test of interest, hereafter called the index test, which is the same in all studies, and a comparator test which may differ between studies. We let $T_{1j}$ denote the index test outcomes in each study and we let $T_{2j}$ denote the comparator test outcomes in each study. In line with previous descriptions (2,3), we define the sensitivity and specificity of the index test in the $j$th study by:

| $\mathrm{logit}{(Se}_{1j})=Pr(T_{1j}=1\vert D=1) =-(\theta_{j} - \alpha_{j}/2)/exp(\beta/2)$  ${logit(Sp}_{1j})=Pr(T_{1j}=0\vert D=0) =(\theta_{j} + \alpha_{j}/2)/exp(-\beta/2)$ | 5 |
| --- | --- |

where $\theta_{j}$ represents the positivity criteria for study $j$. The positivity criteria, or cut-off value, models the dependence between the true positive fraction and false positive fraction in each study. $\alpha_{j}$ represents the diagnostic accuracy and measures the mean difference in test accuracy between individuals ‘diseased’ and individuals ‘non-diseased’ in study $j$. $\beta$ the scale parameter, allows differences in the variation of outcomes between disease positive and disease negative individuals thus allowing asymmetry in the ROC curve. Both $\theta$ and$\alpha$ parameters are modelled as random effects with independent normal distributions to incorporate variation between studies:

| $\theta_{j}\sim N(\Theta, \sigma_{\theta})$  $\alpha_{j} \sim N(\Lambda, \sigma_{\alpha})$ | 6 |
| --- | --- |

Equations 5 and 6 represent the model specification for the full DAT model described in the main text, and it includes no covariates. However, in our sensitivity analyses we also considered heterogeneity between 7 geographic regions where we expected the accuracy of the test to differ. For this we allow the mean of $\theta$ to be a function of a study-level covariate. We let $Z_{\mathrm{mj}}$take the value 1 if the data are from geographic region $m (m=1,..,M)$, and 0 otherwise, where $M$ is the total number of geographic regions considered,. The distribution of $\theta$ and its standard deviation are then assumed to be:

| $\theta_{j}\sim N(\Lambda_{m}Z_{\mathrm{mj}}+\Lambda_{m}Z_{\mathrm{Bj}}+{,\ldots,\Lambda}_{M}Z_{\mathrm{Mj}},\sigma_{\theta})$  $\sigma_{\theta}\sim N(0,1)$ | 7 |
| --- | --- |

where $\Delta_{m}$ represents the coefficient for each geographic region.

We present pooled estimates of sensitivity and specificity which are given by:

| $\mathrm{logit}\left( \mathrm{Pooled}\mathrm{Se}_{1} \right)=-\left( \left( \Theta-\Lambda/2 \right)/\exp\left( \beta/2 \right) \right)$  $logit (Pooled \mathrm{Sp}_{1})=((\Theta+\Lambda/2)/exp(-\beta/2))$ | 8 |
| --- | --- |

And, where we included a covariate, $\Theta$ is replaced by the corresponding coefficient. For example, to estimate the pooled sensitivity and specificity of for geographic region 1:

| $\mathrm{logit}\left( \mathrm{Pooled}{Region 1 Se}_{1} \right)=-\left( \left( \Theta_{1}-\Lambda/2 \right)/\exp\left( \beta/2 \right) \right)$  $logit (Pooled Region 1 \mathrm{Sp}_{1})=((\Theta_{1}+\Lambda/2)/exp(-\beta/2))$ | 9 |
| --- | --- |

Importantly, we also present a prediction of sensitivity and specificity in a new study. Predicted estimates are important because the pooled estimates in a meta-analysis only represent an average estimate among the studies included in the analysis. Predicted estimates on the other hand account for the variation captured through the modelling framework and can be used as priors for sensitivity and specificity of the index test in a new study. We predict sensitivity and specificity by replacing $\Theta$ with $\theta_{newstudy}$ and $\Lambda$ with $\alpha_{newstudy}$:

| $\theta_{newstudy}\sim N\left( \Theta,\sigma_{\theta} \right)$  $\alpha_{newstudy}\sim N\left( \Lambda,\sigma_{\alpha} \right)$ | 10 |
| --- | --- |

**Prior Specification**

Priors for all models are in line with those used by Dendukuri *et al.* (3), where the priors were chosen ensure a uniform distribution for the pooled estimate of sensitivity over 0.5 to 1. We use the following priors: $\Theta$~(0,1.5), $\Lambda$~𝑁(0,2) and, 𝛽~𝑈𝑛𝑖𝑓𝑜𝑟𝑚(−0.75,0.75). Variance parameters $\sigma_{\theta}$ 𝑎𝑛𝑑 $\sigma_{\alpha}$ follow zero-truncated standard normal distributions. We allow prevalence, and sensitivity and specificity of the index test in each study to be uniform over 0 to 1. However for each comparator test, we assume that the probability of a positive test is greater in a disease positive individual than a disease negative individual $(i.e. we set the constraint that {Se}_{2j}>1-{Sp}_{2j})$.

**References:**

1. Rutter CM, Gatsonis CA. A hierarchical regression approach to meta-analysis of diagnostic test accuracy evaluations. Stat Med. 2001;20(19):2865-84.
2. Dendukuri N, Joseph L. Bayesian approaches to modeling the conditional dependence between multiple diagnostic tests. Biometrics. 2001;57(1):158-67.
3. Dendukuri N, Schiller I, Joseph L, Pai M. Bayesian meta-analysis of the accuracy of a test for tuberculous pleuritis in the absence of a gold standard reference. Biometrics. 2012;68(4):1285-93.
