# Supplementary material for: Accuracy of the direct agglutination test for diagnosis of visceral leishmaniasis: a systematic review and meta-analysis
Source: BMC Infect Dis. 2023 Nov 9;23:782. doi: 10.1186/s12879-023-08772-1 (PMC10636880; doi:10.1186/s12879-023-08772-1)
Supplement: Supplementary file 4 — Additional file 4. QUADAS-2 scoring for each study. Studies were scored as high or low risk of bias and applicability. [file 12879_2023_8772_MOESM4_ESM.docx]

Additional file 4. QUADAS-2 scoring for each study. Studies were scored as high or low risk of bias and applicability

| Author | Year | Risk of bias | | | | Applicability | | |
| --- | --- | --- | --- | --- | --- | --- | --- | --- |
|  |  | Patient selection | Index test | Comparator test | Flow and timing | Patient selection | Index test | Comparator test |
| Abass | 2020 | High | Low | Low | Low | Low | Low | Low |
| Abass | 2015 | High | Low | Low | Low | Low | Low | Low |
| Abass | 2007 | Low | Low | Low | Low | Low | Low | Low |
| Abass | 2006 | High | Low | Low | Low | Low | Low | Low |
| Abdalla | 2011 | Low | Low | Low | Low | Low | Low | Low |
| Abdallah | 2004 | High | Low | Low | Low | Low | Low | Low |
| Abera | 2016 | Low | Low | Low | Low | Low | Low | Low |
| Akhoundi | 2010 | High | Low | Low | Low | Low | Low | Low |
| Akhoundi | 2013 | High | Low | Low | Low | Low | Low | Low |
| Al-Nahhas | 2008 | High | Low | Low | Low | Low | Low | Low |
| Asfaram | 2017 | Low | Low | Low | Low | Low | Low | Low |
| Ashkanifar | 2016 | Low | Low | Low | Low | Low | Low | Low |
| Ayelign | 2020 | High | Low | Low | Low | Low | Low | Low |
| Azazy | 2003 | High | High | Low | Low | Low | Low | Low |
| Babiker | 2007 | Low | Low | Low | Low | Low | Low | Low |
| Barbosa Junior | 2015 | Low | High | Low | Low | Low | Low | Low |
| Basiye | 2010 | High | Low | Low | Low | Low | Low | Low |
| Bejano | 2021 | Low | Low | Low | Low | Low | Low | Low |
| Bekele | 2018 | Low | Low | Low | Low | Low | Low | Low |
| Bern | 2000 | High | Low | Low | Low | Low | Low | Low |
| Boelaert | 1999 | Low | Low | Low | Low | Low | Low | Low |
| Boelaert | 1999 | High | Low | Low | Low | Low | Low | Low |
| Boelaert | 2004 | High | Low | Low | Low | Low | Low | Low |
| Canavate | 2011 | High | Low | Low | Low | Low | Low | Low |
| Cavalcanti | 2012 | Low | Low | Low | Low | Low | Low | Low |
| Chakravarty | 2019 | Low | Low | Low | Low | Low | Low | Low |
| Chappuis | 2003 | High | Low | Low | Low | Low | Low | Low |
| Chowdhury | 1991 | High | Low | Low | Low | Low | Low | Low |
| Cummins | 1994 | High | Low | Low | Low | Low | Low | Low |
| Cunha | 2020 | Low | Low | Low | Low | Low | Low | Low |
| Dalimi | 2018 | Low | Low | Low | Low | Low | Low | Low |
| de Assis | 2011 | High | Low | Low | Low | Low | Low | Low |
| de Beer | 1991 | Low | Low | Low | Low | Low | Low | Low |
| De Doncker | 2005 | High | Low | Low | Low | Low | Low | Low |
| de Korte | 1990 | High | Low | Low | Low | Low | Low | Low |
| Deborggraeve | 2008 | High | Low | Low | Low | Low | Low | Low |
| El Harith | 2003 | High | Low | Low | Low | Low | Low | Low |
| El Mutasim | 2006 | Low | Low | Low | Low | Low | Low | Low |
| el Safi | 1989 | High | Low | Low | Low | Low | Low | Low |
| el-Masum | 1995 | Low | Low | Low | Low | Low | Low | Low |
| El-Moamly | 2011 | High | Low | Low | Low | Low | Low | Low |
| El-Safi | 2003 | High | High | Low | Low | Low | Low | Low |
| El-Safi | 1991 | Low | High | Low | Low | Low | Low | Low |
| Fakhar | 2014 | Low | Low | Low | Low | Low | Low | Low |
| Fakhar | 2008 | Low | Low | Low | Low | Low | Low | Low |
| Fakhar | 2011 | Low | Low | Low | Low | Low | Low | Low |
| Gavgani | 2007 | Low | High | Low | Low | Low | Low | Low |
| Ghasemian | 2014 | High | Low | Low | Low | Low | Low | Low |
| Gidwani | 2009 | Low | High | Low | Low | Low | Low | Low |
| Hailu | 2006 | Low | Low | Low | Low | Low | Low | Low |
| Sharmin | 2019 | Low | Low | Low | Low | Low | Low | Low |
| Islam | 2004 | High | Low | Low | Low | Low | Low | Low |
| Jacquet | 2006 | High | Low | Low | High | Low | Low | Low |
| Johanson | 2020 | High | Low | Low | High | Low | Low | Low |
| Khanal | 2010 | Low | Low | Low | Low | Low | Low | Low |
| Kilic | 2008 | Low | Low | Low | Low | Low | Low | Low |
| Manomat | 2017 | Low | Low | Low | Low | Low | Low | Low |
| Mansour | 2007 | High | Low | Low | Low | Low | Low | Low |
| Mbati | 1999 | High | Low | Low | Low | Low | Low | Low |
| Mohamed | 2019 | Low | Low | Low | Low | Low | Low | Low |
| Nigro | 1996 | Low | Low | Low | Low | Low | Low | Low |
| Okong'o-Odera | 1993 | High | Low | Low | Low | Low | Low | Low |
| Osman | 2016 | High | Low | Low | Low | Low | Low | Low |
| Pal | 2004 | Low | High | Low | Low | Low | Low | Low |
| Pedras | 2008 | High | Low | Low | Low | Low | Low | Low |
| Ritmeijer | 2006 | High | Low | Low | Low | Low | Low | Low |
| Sarkari | 2015 | Low | Low | Low | Low | Low | Low | Low |
| Schallig | 2002 | High | Low | Low | Low | Low | Low | Low |
| Seaman | 1992 | Low | Low | Low | Low | Low | Low | Low |
| Semiao-Santos | 1995 | Low | Low | Low | Low | Low | Low | Low |
| Silva | 2005 | High | Low | Low | Low | Low | Low | Low |
| Singh | 2018 | High | Low | Low | High | Low | Low | Low |
| Topno | 2010 | Low | Low | Low | Low | Low | Low | Low |
| Vallur | 2014 | Low | Low | Low | High | Low | Low | Low |
| Veeken | 2003 | Low | Low | Low | Low | Low | Low | Low |
| Zijlstra | 1991 | Low | Low | Low | Low | Low | Low | Low |
| Zijlstra | 2001 | High | Low | Low | Low | Low | Low | Low |
